# One-Step Microwave Synthesis of NiSb/NiSe Nanomaterials for High Performance Supercapacitors

**DOI:** 10.3390/molecules30102168

**Published:** 2025-05-15

**Authors:** Qianwen Duan, Hongjie Kang, Cuiling Guan, Xueming Zhao, Haidong Zhao, Buqin Jing, Zhen Lu, Feng Feng

**Affiliations:** School of Chemistry and Chemical Engineering, Shanxi Datong University, Datong 037009, China; 18834839312@163.com (Q.D.); 15603450662@163.com (H.K.); dtdxgcl@163.com (C.G.); m19503425574@163.com (X.Z.); zhaohd2004@126.com (H.Z.)

**Keywords:** NiSb/NiSe, microwave method, supercapacitors

## Abstract

This paper investigated the preparation of NiSb/NiSe nanomaterials using a microwave method and explored their electrochemical properties and potential applications in supercapacitors. The NiSb/NiSe nanomaterials were synthesized on nickel foam using microwave radiation, resulting in uniformly distributed flower-like nanostructures. This structure not only provided abundant electrochemical reaction sites, but also improved the electrical conductivity and ion diffusion, contributing to the overall performance of supercapacitors. Electrochemical tests showed that the NiSb/NiSe material exhibited a high specific capacity of 525 mAh g^−1^ at 1 A g⁻^1^ and maintained 65% capacity after 8000 cycles, demonstrating excellent cycling stability and battery-type charge storage capability. In addition, a hybrid supercapacitor assembled using NiSb/NiSe as the anode material and activated carbon (AC) as the cathode material achieved an energy density of 100.34 Wh kg^−1^ at a power density of 774.9 W kg^−1^, significantly enhancing energy storage efficiency. The effect of different microwave powers and reaction times on the morphology and electrochemical properties of the materials were further investigated, with the optimal preparation conditions found to be 800 W and 150 s. The NiSb/NiSe materials synthesized under this condition not only have the best electrochemical properties, but also exhibit low charge transfer impedance and excellent electrical conductivity. In summary, NiSb/NiSe flower-like nanomaterials as supercapacitor electrode materials demonstrate great potential for energy storage applications due to their high specific capacity, good cycling stability and high energy density.

## 1. Introduction

The excessive use of fossil fuels has led to increasingly severe environmental pollution issues, including greenhouse gas emissions, air pollution, and ecosystem degradation. Addressing these urgent environmental challenges requires the development of renewable and clean energy sources, which can reduce dependence on fossil fuels and their consumption. Consequently, novel energy storage devices are becoming increasingly crucial in the ongoing energy transition [1,2,3]. Supercapacitors (SCs), a type of large-scale energy storage device, have become a focal point of contemporary research. However, due to limitations in electrode materials, supercapacitors cannot meet the growing demand, making the development of high-performance electrode materials for supercapacitor energy storage an urgent priority [4,5,6].

Efforts have been made to identify high-performance electrode materials, including carbon materials [7,8], alloy materials [9], metal oxides [10], and metal sulfides [11]. Nickel-based materials, with their abundant reserves, low cost, and diverse valence states, possess inherent advantages in energy storage applications. Selenium, positioned between metallic and nonmetallic elements, is a natural semiconductor with excellent electrical conductivity. NiSe nanorod arrays on nickel foam were synthesized by Tian’s group using a hydrothermal method, achieving a specific capacitance of 6.81 F cm^−2^ and retaining capacitance after 3000 cycles [12]. However, single NiSe electrode materials are susceptible to structural damage during charge-discharge cycles, leading to material degradation and detachment, which results in unsatisfactory overall performance and cycling stability when used as an electrode material [13].

It has been demonstrated that combining NiSe with carbon or other metal materials with a large specific surface area could exploit the synergistic effect between the metals to alleviate the mechanical stress induced by volume changes during the charge-discharge process. This is an effective method for enhancing the electrochemical performance and cycling stability of electrode materials [14]. For example, Kirubasankar’s group successfully synthesized nickel foam-supported NiSe-G composites using a hydrothermal method. Scanning electron microscopy (SEM) revealed that NiSe nanoparticles were uniformly distributed on graphene nanosheets, yielding a specific capacitance of 1280 F g^−1^ at 1 A g^−1^, with a capacity retention rate of 98% after 2500 cycles [15]. The significant increase in specific capacitance of the NiSe-G composite was attributed to the additional electrochemically active sites provided by graphene nanosheets. Binary metal selenides (Ni-Co-Se) were synthesized by Xie et al., yielding a specific capacitance of 1494.9/1192.4 F g^−1^ at 1/20 A g^−1^ with a retention rate of 79.8%. Additionally, 81.5% of the initial specific capacitance was retained after 10,000 cycles at 10 A g^−1^ [16]. This excellent performance was attributed to the synergistic interaction among the three metals, resulting in a unique microstructure and high electrical conductivity. Saranya et al. synthesized spherical Ni-Mn diselenide nanoparticles with varying compositions using a simple hydrothermal method. Ni_0.75_Mn_0.25_Se_2_ exhibited good electrochemical properties with a specific capacitance of 1490 F g^−1^ [17].These studies demonstrated that the incorporation of other elements can enhance the electrochemical properties of NiSe.

Elements such as cobalt (Co) and manganese (Mn) are complemented by antimony (Sb), a metallic element from group VA, which is abundantly available in the Earth’s crust and is considered an ideal candidate for enhancing electrochemical activity [18,19,20]. Sb has been reported to modulate the conductivity and capacitive characteristics of materials. For instance, its doping in SnO_2_ has been shown to enhance capacitive properties, resulting in a specific capacitance of 158.2 F g^−1^ [21]. Similarly, Yude Wang et al. synthesized Sb-doped SnO_2_ materials, which demonstrated improved capacitance characteristics in electrochemical tests [22]. Ali et al. investigated Co_3_O_4_ doped with varying Sb contents, showing that Sb doping significantly enhanced the electrochemical properties of Co_3_O_4_ nanosheets, with a capacity retention of 82.02% after 3000 cycles [23]. In 2020, Mariappan’s group prepared antimonene/3D Ni nanostructures through a chemical deposition technique. The electrodes exhibited an ultra-high specific capacitance of 6854.45 F g^−1^. When assembled into asymmetric supercapacitor (ASC) devices, they achieved an impressive energy density of 84.79 Wh kg^−1^ and a power density of 20,625 W kg^−1^ [24]. Despite their potential for electrochemical energy storage applications, challenges remain in developing cost-effective, large-scale methods for Sb nanosheet preparation and in preventing their aggregation.

To achieve this purpose, the incorporation of antimony (Sb) into nickel selenide (NiSe) results in a synergistic enhancement of properties through the formation of a complex that consists of nickel selenide and nickel antimonide. The introduction of Sb improves the electrical conductivity of the material, provides abundant chemical reaction sites, and reduces the migration pathways for electrons and ions. This study presents NiSb/NiSe floral nanomaterials as high-performance electrode materials for supercapacitors. NiSb/NiSe nanomaterials were prepared on nickel foam using a short-duration, high-stability microwave method, which can be tuned for the morphology of the material by varying the power and time of microwave radiation. The NiSb/NiSe anode achieved a specific capacity of 525 mAh g^−1^ at 1 A g^−1^ and retained 65% of its initial capacity after 8000 cycles. The asymmetric supercapacitor, assembled with activated carbon (AC) as the cathode, attained an energy density of 100.34 Wh kg^−1^ and a power density of 774.9 W kg^−1^, maintaining 68% of its initial capacity after 20,000 cycles, thereby demonstrating excellent electrochemical performance and good cycling stability.

## 2. Results

### 2.1. Morphology and Microstructure

**Figure 1:**
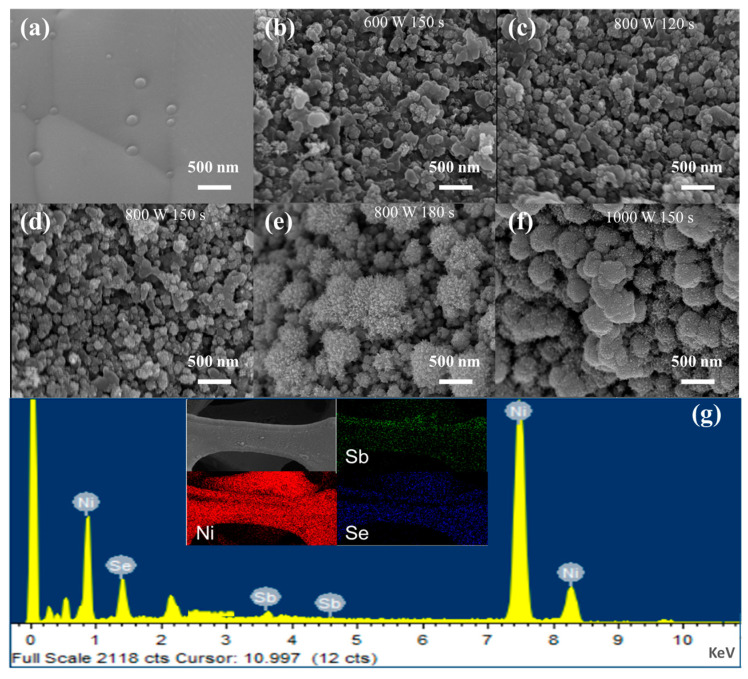
SEM images and EDS pattern of NiSb/NiSe electrode material: (**a**) the blank nickel foam; (**b**–**f**) products prepared at 600 W 150 s, 800 W 120 s, 800 W 150 s, 800 W 180 s and 1000 W 150 s respectively; (**g**) the electronic energy pattern of the product prepared at 800 W 150 s. The electronic energy pattern of NiSb/NiSe electrode material were shown in the following table.

To attain uniform morphological characteristics and enhanced electrochemical activity, the microwave power and reaction time were systematically investigated and optimized. As summarized in Table 1, sample A3 (800 W, 150 s) demonstrated the highest specific capacity of 525 mAh g^−1^, which can be attributed to its well-defined, uniformly dispersed flower-like nanostructure (Figure 1d). In contrast, lower microwave power (600 W) or shorter reaction time (120 s) led to incomplete nanostructure growth (samples A1 and A2), whereas excessive power (1000 W) or prolonged duration (180 s) induced particle agglomeration (samples A4 and A5), resulting in diminished electrochemically active sites. These findings conclusively demonstrate that 800 W for 150 s constitutes the optimal synthesis condition for achieving balanced nucleation and growth kinetics.

The microscopic morphology of NiSb/NiSe was examined using scanning electron microscopy (SEM), revealing the self-growth of NiSb/NiSe nanoparticles on nickel foam, with an overall morphology characterized by the distribution of nanoball particles. The microscopic morphology of blank nickel foam was presented in Figure 1a. The preparation conditions depicted in Figure 1b–d were 600 W for 150 s, 800 W for 120 s, and 800 W for 150 s. The figures indicated that as microwave power and reaction time increased, the product particles became more homogeneous, with those prepared at 800 W for 150 s exhibiting a size of approximately 100 nm. Further increase in microwave heating time or power resulted in significant changes in product size, reaching approximately 500 nm with a flower-like distribution (Figure 1e,f). The size and microscopic morphology of NiSb/NiSe particles can be regulated by microwave power and time, both of which significantly influence electrochemical performance. Uniform morphology was essential for achieving superior electrochemical performance. The highest capacity value was achieved with a uniform nanosphere morphology at 800 W for 150 s. Energy dispersive spectroscopy (EDS) plots confirmed the successful self-growth of NiSb/NiSe complexes on nickel foam. The complexation of NiSb and NiSe enhanced the number of electrically active sites and improved conductivity, thereby accelerating electron and ion migration. Similarly, EDS plots demonstrated the simultaneous presence of Ni, Sb, and Se, confirming the successful preparation of the composite electrode material.

**Figure 2:**
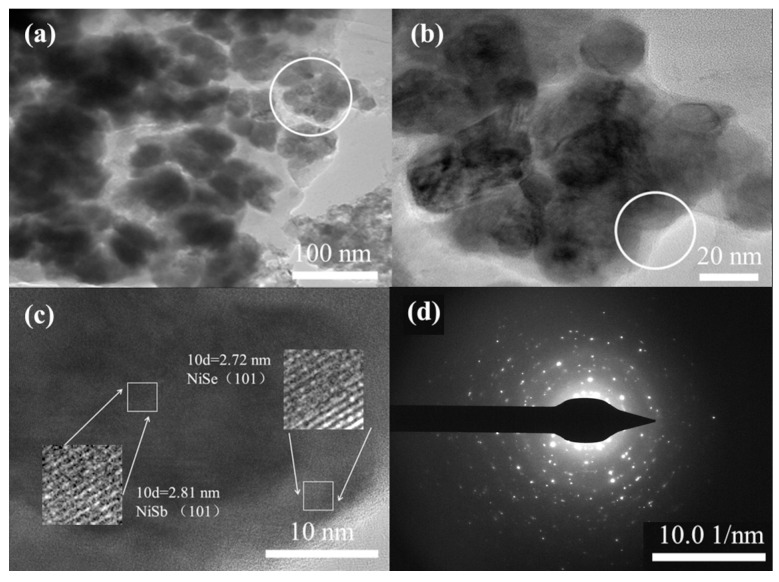
TEM images of NiSb/NiSe electrode material: (**a**) TEM image; (**b**,**c**) HRTEM images; (**d**) selected area electron diffraction (SAED) image.

After sonication of NiSb/NiSe nickel foam in anhydrous ethanol for 15 min and drying of the solution, the internal morphology and structure of the samples were investigated using transmission electron microscopy tests, and it could be seen from Figure 2 that NiSb/NiSe nanoparticles with 100 nm size were agglomerated from smaller particles (30–50 nm). Figure 2b showed a magnification of the nanoparticles circled in Figure 2a, further confirming the agglomerate structure. Figure 2c showed a high-resolution enlargement of the circled portion in Figure 2b, where the (101) crystal faces of NiSe and NiSb appeared in the same small grains, indicating that the composite of the two is not a mixing of the atomic level, but rather, the small grains were formed separately, and then the small grained randomly rotate to align and combine during the growth process, which was consistent with the OA growth mechanism of crystal growth (oriented attach) [25]. This result was consistent with the XRD results, where diffraction peaks from the (101) crystal faces of both NiSe and NiSb appeared in the spectra. In Figure 2d, the grains were analyzed by Selected Area Electron Diffraction (SAED). The SAED pattern showed diffraction rings formed by scattered diffraction spots, confirming the polycrystalline nature of the product.

**Figure 3:**
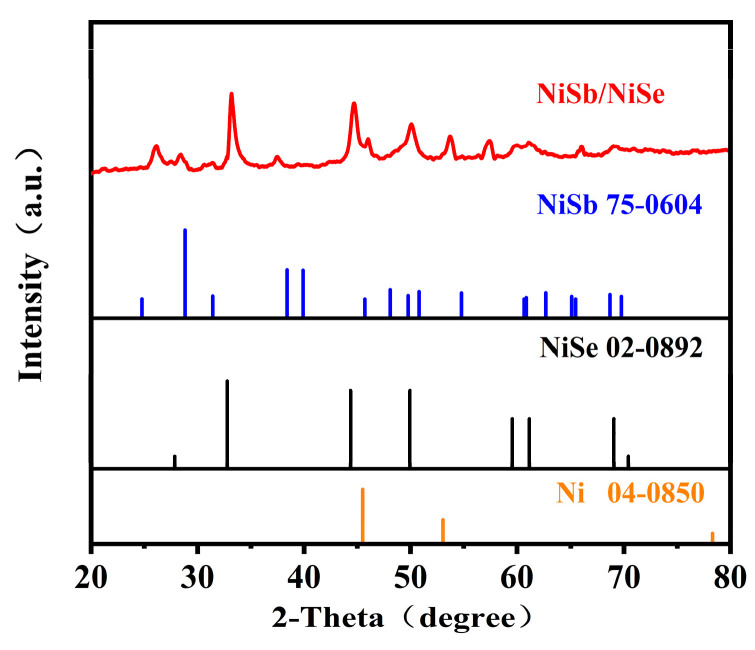
XRD patterns of NiSb/NiSe electrode material.

The crystal structure of the prepared NiSb/NiSe was further analyzed using X-ray powder diffraction (XRD) tests and the results were presented Figure 3. The diffraction peaks at 31.7°, 46.53°, 54.27°, 57.44°, 66.38°, and 76.93° corresponded to the (101), (110), (200), and (202) crystal planes of NiSb (JCPDS no. 75-0604), while the diffraction peaks at 32.78°, 59.55°, and 61.16° were attributed to the (101), (102), (103), (110), and (201) crystal faces of NiSe (JCPDS no. 02-0892). The three peaks (44.5°, 51.8° and 76.4°) corresponded to the crystal planes of the cubic phase Ni substrate (JCPDS no. 04-0850). The XRD results showed that the NiSb/NiSe composite electrode materials were successfully prepared. The diffraction peak at 38.2° may be due to trace impurities.

**Figure 4:**
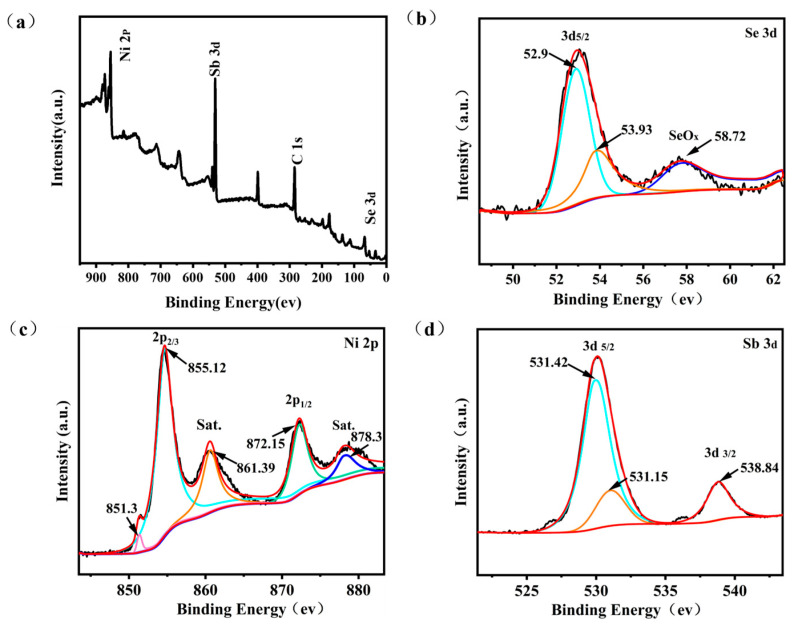
(**a**) XPS survey spectrum; XPS elemental spectra of (**b**) Se 3d (**c**) Ni 2p (**d**) Sb 3d of NiSb/NiSe electrode material.

The elemental valence and composition of NiSb/NiSe were further analyzed using XPS. Figure 4a showed the XPS full spectrum of NiSb/NiSe electrode material, indicating that the sample was composed of Ni, Se, Sb, and C elements. In the Se 3d spectrogram (Figure 4b), the Se 3d_5/2_ peak was located at 52.9 eV, and the 3d_3/2_ peak was located at 53.93 eV, which was consistent with the Ni-Se bonding in nickel selenides. In addition, the appearance of the peak at 58.72 eV indicated the possible presence of SeO_X_, which may be due to the surface oxidation of Se. Figure 4c showed the high-resolution spectrum of Ni 2p. From the results of the peak deconvolution, it could be seen that the satellite peaks were located at 878.3 eV and 861.39 eV. The pronounced satellite intensity likely arose from plasmonic interactions in the nanostructured composite. The peaks corresponding to Ni^2^⁺ in NiSe_2_ were at 872.15 eV and 855.12 eV. In addition, the peaks at 851.3 eV corresponded to the metallic Ni of the NF substrate. In Figure 4d, the high-resolution spectrum of the Sb element showed that the peaks appearing at the binding energies of 538.84 eV and 531.42 eV corresponded to the chemical state of Sb 3d, which proved that the composite material was related to Sb_2_O_5_. In addition, a new peak appeared at the binding energy of 531.15 eV, indicating the formation of some Sb-based by-products. The XPS pattern of NiSb/NiSe electrode confirmed the presence of the elements Ni, Sb and Se. The presence of these oxide species may significantly influence the electrochemical performance of the material. On one hand, the existence of NiSe_2_ could partially participate in redox reactions, thereby modulating the capacitive characteristics of the material. On the other hand, Sb_2_O_5_ and other Sb-based oxides may alter the electron conduction pathways and the distribution of active sites, thus affecting the charge transfer process.

### 2.2. Electrochemical Performance Analysis of NiSb/NiSe Electrode Materials

#### 2.2.1. Electrochemical Properties of the Three-Electrode System

**Figure 5:**
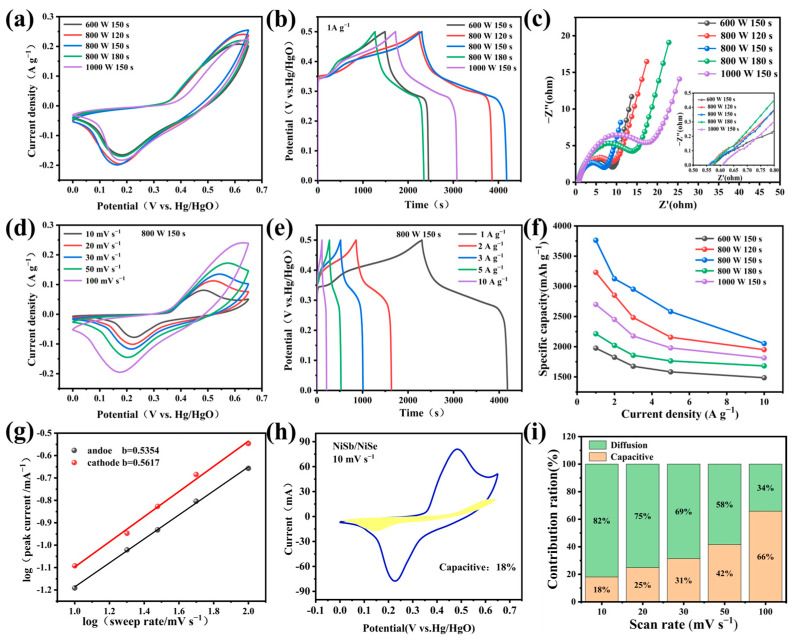
(**a**) CV curves of NiSb/NiSe under different microwave conditions; (**b**) GCD curves for different reaction parameters; (**c**) Nernquist diagram of NiSb/NiSe under different reaction parameters; (**d**) CV curve of NiSb/NiSe under optimal conditions (800 W/150 s); (**e**) GCD curve of sample A3; (**f**) The specific capacity of NiSb/NiSe samples under different preparation conditions; (**g**) NiSb/NiSe charge storage dynamics calculation; (**h**) Capacity share of NiSb/NiSe energy storage at 10 mV^−1^; (**i**) Relative contribution of capacity and diffusion-controlled charge storage.

The electrochemical performance of NiSb/NiSe electrodes was tested in the three-electrode system with 6 M KOH under different conditions. Figure 5a showed the CV curves of NiSb/NiSe materials under different reaction conditions, and the mathematical integral area of the CV curves of sample A3 (800 W 150 s) was the largest, while the integral area of sample A1 (600 W 150 s) was the smallest, which may be due to the long time resulting in the electrode material being burned. The microwave power and reaction time significantly influenced the electrochemical performance of NiSb/NiSe. Figure 5b showed the GCD curves of NiSb/NiSe under different conditions, confirming that sample A3 exhibited the longest discharge time and the highest specific capacity. The capacity values for samples A1, A2, A3, A4, and A5 were 264 mAh g^−1^ 450 mAh g^−1^, 525 mAh g^−1^, 306 mAh g^−1^, and 378 mAh g^−1^, respectively. The performance variation originated from time-dependent morphological evolution: Shorter durations (120 s, A2) promoted nucleation but limited growth, producing undersized particles with constrained surface area (Figure 1c). Optimal 150 s irradiation (A3) generated hierarchical flower-like nanostructures (Figure 1d) with enhanced active site density and ion diffusion kinetics. Prolonged exposure (180 s, A4) induced particle agglomeration (Figure 1e), causing pore blockage and increased charge transfer resistance (evidenced by Nyquist plot analysis in Figure 5c), ultimately degrading capacity. These results highlight the critical requirement for precise temporal control in microwave-assisted synthesis to optimize nucleation-growth equilibrium. The differences in the internal resistance of the samples under different reaction conditions were shown in Figure 5c, and the sample A3 exhibited a smaller solution resistance in the low-frequency region compared to the materials under other conditions. It can also be seen from the figure that the semicircle diameter of sample A3 was the smallest, indicating that the charge transfer internal resistance of the electrode surface was also the smallest, which aligns with its highest specific capacity (525 mAh g^−1^) in Figure 5f. For other samples (e.g., A4 and A5), larger Rct (bigger semicircles) correlate with lower capacities due to excessive particle agglomeration, which hinders ion diffusion and reduces active sites. The variation of the CV curves of sample A3 (800 W 150 S) at different scan rates was shown in Figure 5d, and the obvious oxidation and reduction peaks indicated the battery-type nature of the material. Figure 5e shows the GCD curve of sample A3. According to the calculation, the capacity of NiSb/NiSe material is 525 mAh g^−1^ at a current density of 1 A g^−1^. Figure 5f showed the line graphs of the capacity values of NiSb/NiSe under different conditions at different current densities, regardless of the conditions, 800 W, 150 S has the highest capacity value. To further elucidate the charge storage mechanism of NiSb/NiSe, the b-values of the electrode material calculated from Equation (1) are 0.5354 and 0.5617 (Figure 5g), both approaching 0.5, indicating a predominantly diffusion-controlled battery-type behavior. As shown in Figure 5h, the surface-controlled capacitive contribution accounted for 18% of the total capacity at 10 mV s^−1^ (yellow region), while the diffusion-controlled contribution increased to 82% (Figure 5i). This arose from the flower-like architecture, which facilitates deeper ion penetration into the electrode bulk, activating volume-based redox reactions between Ni^2+^/Ni^3+^ and Sb^3+^/Sb^5+^-a hallmark of efficient battery-type charge storage. This phenomenon originated from the flower-like nanostructure (Figure 1d): at high scan rates, ions can only access the particle surface for rapid charge transfer (capacitive dominance); at low scan rates, ions penetrate the porous architecture, activating bulk redox reactions (enhanced diffusion contribution). These results demonstrated that the charge storage in NiSb/NiSe arises from the synergy between surface capacity and bulk diffusion, with its unique nanoarchitecture providing an ideal platform to balance both mechanisms.

I = a × v^b^ (1)

**Figure 6:**
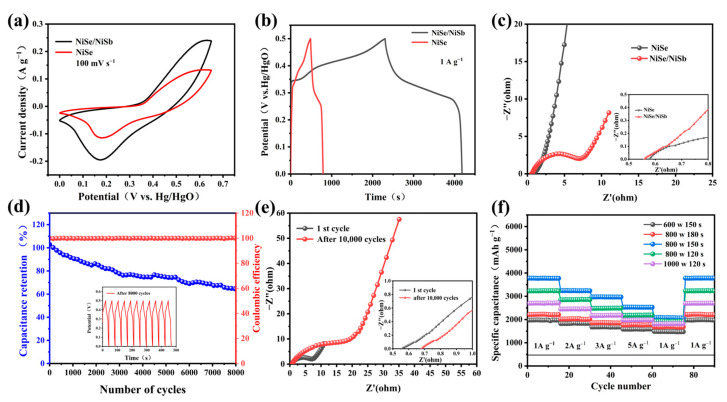
(**a**) CV diagrams of NiSb/NiSe and NiSe; (**b**) GCD curves of NiSb/NiSe and NiSe; (**c**) EIS charts for NiSb/NiSe and NiSe; (**d**) cycle stability and coulomb efficiency; (**e**) EIS maps before and after the cycle; (**f**) magnification performance chart.

NiSb/NiSe composite electrode materials were prepared by microwave method, and the introduction of Sb greatly enhanced the electrochemical performance of NiSe. Figure 6 showed the CV, CP and EIS of NiSe and NiSb/NiSe complexes, respectively. From Figure 6a at 100 mV s^−1^, there was a clear difference between the CV plots of the two. The integral area of the curve was larger for NiSb/NiSe, as can be seen in the plot of Figure 6b, where the constant current charge/discharge curve of NiSb/NiSe reached 525 mAh g^−1^ (1 A g^−1^), compared to the maximum value of specific capacity of NiSe of 78.3 mAh g^−1^. The EIS impedance plots in Figure 6c clearly showed that the Rs of composite NiSb/NiSe and monolithic NiSe were 0.56 and 0.58 Ω. As shown in Figure 6d, NiSb/NiSe displayed excellent cycling stability, with capacity retention of more than 67% after the cycling performance test of continuous charge/discharge of 8000 turns at 10 A g^−1^. This fully indicated that the introduction of nanostructured NiSb attenuated the mechanical stress caused by the volume change during repeated charging and discharging and greatly enhanced the service life of the electrode material. In Figure 6e, after 8000 cycles, the electrode structure lost part of the active material and part of the electrode structure might be damaged, which led to the degradation of the performance and a slight increase of the internal resistance. The multiplicative performance of NiSb/NiSe was shown in Figure 6f, where the capacity value of the sample A3 was maintained at 525 mAh g^−1^ under 1 A g^−1^ after 15 charge/discharge cycles each at different current densities, showing a good multiplicative electrochemical performance. good multiplicative electrochemical performance. The comparison of NiSb/NiSe with other materials of the same type was shown in Table 2.

#### 2.2.2. Electrochemical Properties of the Two-Electrode System

**Figure 7:**
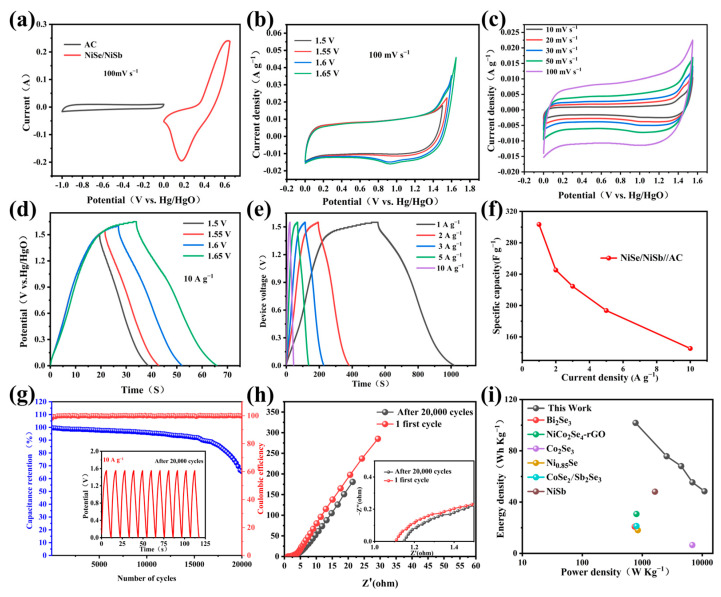
(**a**) CV plots of NiSb/NiSe and AC; (**b**) CV diagrams at different voltages; (**c**) CV map of NiSb/NiSe//AC at different scanning rates at 1.6 V; (**d**) GCD curves at different voltages (10 A g^−1^); (**e**) GCD curve of NiSb/NiSe//AC AC at different currents; (**f**) Line chart of current GCD curve of NiSb/NiSe//AC at different voltages; (**g**) cycle stability and coulomb efficiency; (**h**) EIS maps before and after 20,000 cycles; (**i**) Energy-power density diagram of different devices.

To further explore the performance of NiSb/NiSe in device applications, an SC device consisting of NiSb/NiSe and an AC cathode was prepared. Figure 7 showed the electrochemical characterization of asymmetric SC (ASC). Figure 7a showed the CV curves of NiSb/NiSe (positive electrode) and AC (negative electrode) at 50 mV s^−1^. NiSb/NiSe exhibited distinct redox peaks (battery-type behavior) within 0–0.6 V, while AC showed near-rectangular double-layer capacitive characteristics in −1.0–0 V, confirming the feasibility of asymmetric device design through complementary potential windows. Figure 7b (voltage optimization) revealed significant polarization (current surge at 1.6 V), indicating electrolyte decomposition or side reactions, which defined 1.55 V as the safe operating window Figure 7c displayed CV curves at 1.55 V under varied scan rates (10–100 mV s^−1^). The quasi-rectangular shape retention at high rates demonstrated excellent rate capability and rapid charge transfer, attributed to the hierarchical porosity of NiSb/NiSe (Figure 1d) and AC. Figure 7d showed the GCD curves of NiSb/NiSe//AC at different voltages and 10 A g^−1^ current densities, and the CV curves showed significant polarization at 1.6 V, so the optimum operating voltage was 1.55 V. Figure 7e quantified the rate performance: a specific capacity of 125.4 mAh g^−1^ (at 1 A g⁻^1^) with 64.8% retention (81.3 mAh g^−1^ at 10 A g⁻^1^), benefiting from the synergistic effects between NiSb/NiSe’s flower-like structure and AC’s high surface area. Figure 7g (cycling stability) showed 68% capacity retention after 20,000 cycles, with Coulombic efficiency > 97%. Capacity fading originated from partial structural collapse of NiSb/NiSe and irreversible oxidation of functional groups on AC. Figure 7h (EIS) demonstrated increased solution resistance and charged transfer resistance, confirming interfacial degradation during cycling. Figure 7i (Ragone plot) highlighted the device’s superior energy density (100.34 Wh kg^−1^ at 774.9 W kg^−1^), and with Table 2, the materials outperforming reported NiSe-based asymmetric supercapacitors [30,31,32,33,34,35].

## 3. Materials and Methods

### 3.1. Material Preparation

Nickel foam (NF) was purchased from Longshengbao Products Company (Guangzhou, China). Before the experiments, the NF was ultrasonically cleaned with hydrochloric acid, ethanol and ultrapure water to ensure that it was free of impurities. It was then dried under vacuum at 80 °C. Selenium powder and antimony trichloride (SbCl_3_) were purchased from Aladdin Industrial Company. Nickel chloride (NiCl_2_) was obtained from Leyan (Shanghai, China). Anhydrous ethanol, ethylene glycol (EG), ethylenediamine (EDA) and potassium hydroxide (KOH) were purchased from Tianjin Damao Chemical Company (Tianjin, China).

### 3.2. Preparation of NiSb/NiSe

**Figure 8:**
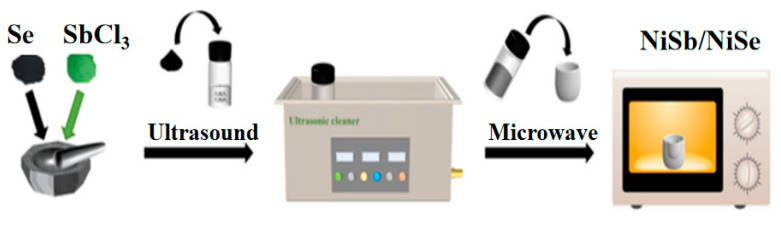
Preparation process of NiSb/NiSe.

15 mg of selenium powder, 13 mg of nickel chloride powder and 3 mg of antimony chloride powder, 300 μL of potassium hydroxide (6 M), 2 mL of C_2_H_8_N_2_ and 1 mL (CH_2_OH)_2_ were dissolved by stirring. The nickel foam was then immersed and after sonication for 15 min, the immersed nickel foam was transferred to a heat-resistant crucible (10 mL). After being kept in a microwave oven at a certain power and time, the NF was washed with deionized water and anhydrous ethanol and further dried (80 °C, 12 h). The loading mass of the active substance is the mass difference of the nickel foam before and after the reaction. The preparation process is shown in Figure 8, and the different reaction conditions are shown in Table 2.

### 3.3. NiSb/NiSe/Ni//AC Assembly of Asymmetric Supercapacitors ASC

A NiSb/NiSe/Ni//AC device was assembled using a NiSb/NiSe electrode for the positive electrode and a 1 × 1 cm^2^ commercial activated carbon (AC) electrode for the negative electrode and tested in 6 M KOH.

### 3.4. Characterization of NiSb/NiSe

The crystal structure of the obtained samples was characterized by powder x-ray diffraction (XRD, Tokyo, Japan). The surface morphology and micro-internal structure of the samples were determined by scanning electron microscopy (SEM, Brno, Czech Republic) and transmission electron microscopy (TEM, Hillsboro, OR, USA). The elemental composition and distribution of the samples were characterized using x-ray photoelectron spectroscopy (XPS, Waltham, MA, USA) and energy dispersive spectroscopy (EDS, Mahwah, NJ, USA).

### 3.5. Electrochemical Testing

Bulleted lists look like this: The electrochemical properties of NiSb/NiSe composites in 6 M potassium hydroxide were tested by cyclic voltammetry (CV), constant current charge/discharge (GCD) and electrochemical impedance method (EIS) was measured at open-circuit potential with a 10 mV AC perturbation over 10^−5^–10^−2^ Hz. A platinum sheet was used as the counter electrode and a mercuric oxide electrode was used as the reference electrode. An electrochemical workstation (CHI660E, Shanghai, China) was used for electrochemical data testing. The cycling stability of the materials was evaluated using the Landt battery test system (CT2001A, Wuhan, China). Capacity values were calculated using the following equation.

C (F g^−1^) = I∆t/(m∆V) (2)

C (mA h g^−1^) = I ∆t/3.6 (3)

In order to further demonstrate the feasibility of the materials to construct high-performance ASCs in KOH electrolyte. A two-electrode test system was assembled with AC as the negative electrode and the synthesized material as the positive electrode. In this case, the mass relationship between the positive and negative electrodes was determined by the following equation:

m^+^⁄m^−^ = C^−^ ΔV^−^/C^+^ ΔV^+^ (4)

Energy density and power density are two key parameters for evaluating the performance of batteries, capacitors and other energy storage systems. They describe the ability of the system to store and release energy and the rate at which energy is delivered per unit of time. Energy density (E) and power density (P) are calculated using the following equations.

E = C ∆V2/7.2(5)

P = 3600 E/∆t (6)

## 4. Conclusions

In this study, NiSb/NiSe electrodes without additives and binder were successfully prepared by a simple microwave method and applied to supercapacitors. The flower-like structure of the material not only provided a large surface area, but also promoted the mass transfer of OH^−1^ ions. In addition, NiSb/NiSe showed additional synergistic effects originating from the combined redox reactions of NiSb and NiSe. As a result, the NiSb/NiSe electrode exhibited a significantly enhanced specific capacity (525 mAh cm^−2^ at 1 A g⁻^1^), characteristic of battery-type energy storage and excellent cycling stability (65% retention after 8000 cycles). In addition, the NiSb/NiSe hybrid supercapacitor exhibited an excellent energy density of 100.34 Wh kg^−1^ at a power density of 774.9 W kg^−1^ in a two-electrode system. The results suggest that NiSb/NiSe nanoporous electrodes under 800 W 150 s microwave radiation can serve as a promising architecture, and the material opens the door to the rational creation of new and promising nanostructured materials for supercapacitors and other energy applications.

## Figures and Tables

**Table 1:** NiSb/NiSe under different reaction conditions.

Sample	Microwave Power	Reaction Time/s	Capacity Value/mAh g^−1^ (1 A g^−1^)
A1	600 W	150 s	264 mAh g^−1^
A2	800 W	120 s	450 mAh g^−1^
A3	800 W	150 s	525 mAh g^−1^
A4	800 W	180 s	306 mAh g^−1^
A5	1000 W	150 s	378 mAh g^−1^

**Table 2:** Comparison of electrochemical properties of NiSb/NiSe with other materials.

Materia	Method	Specific Capacity/mAh g^−1^	Cyclic Stability/Cycles	Ref.
NiSe-G	Hydrothermal	142.2 mAh g^−1^	98%/2500	[15]
NiSe@Mn	Hydrothermal	80.1 mAh g^−1^	14%/1400	[18]
NiSe@MoSe_2_	Hydrothermal	128.2 mAh g^−1^	65%/1000	[26]
NiCo_2_Se	Hydrothermal	60.1 mAh g^−1^	90%/1000	[27]
NiSe@ZnSe	Hydrothermal	61.4 mAh g^−1^	99%/2000	[28]
NiSe_2_@C	High temperature calcination	111.5 mAh g^−1^	94%/1000	[29]
NiSb/NiSe	Microwave	525 mAh g^−1^	65%/8000	This work

## Data Availability

The data presented in this study are available on request from the corresponding author.
